# Regenerative Surgery, State of the Art and New Perspectives: A Narrative Review

**DOI:** 10.3390/medicina62030432

**Published:** 2026-02-25

**Authors:** Federica Pulicari, Matteo Pellegrini, Sabrina Darwish, Anita Groppi, Massimo Porrini, Moreno Bosotti, Margherita Rossi, Francesco Spadari

**Affiliations:** 1 Maxillo-Facial and Odontostomatology Unit, Fondazione IRCCS Ca’ Granda Ospedale Maggiore Policlinico, University of Milan, 20122 Milan, Italy; matteo.pellegrini@unimi.it (M.P.); sabrina.darwish@studenti.unimi.it (S.D.); anita.groppi01@universitadipavia.it (A.G.); massimo.porrini@unimi.it (M.P.); moreno.bosotti@unimi.it (M.B.); margherita.rossi1@unimi.it (M.R.); francesco.spadari@unimi.it (F.S.); 2 Department of Biomedical, Surgical and Dental Sciences, University of Milan, Via della Commenda 10, 20122 Milan, Italy; 3 Section of Dentistry, Department of Clinical, Surgical, Diagnostic and Pediatric Sciences, University of Pavia, 27100 Pavia, Italy

**Keywords:** oral surgery regeneration, laser therapy, photobiomodulation, mesenchymal stem cells, platelet-rich plasma (PRP), 3D-printed scaffolds, biomaterials, growth factors, bone regeneration, regenerative oral surgery

## Abstract

*Background and Objectives:* Soft tissue regeneration in oral surgery has undergone remarkable progress in the last decade, supported by the development of innovative laser technologies, advanced biomaterials, platelet-rich plasma (PRP), mesenchymal stem cells (MSCs), and three-dimensional (3D) printing. Lasers are increasingly used not only for incision and coagulation but also for photobiomodulation, promoting cellular proliferation, angiogenesis, and tissue healing. The purpose of this review is to analyze the current advances in soft tissue regeneration, with a particular focus on the clinical use of lasers and their integration with other regenerative strategies. In parallel, hard tissue regeneration has evolved through the synergistic use of bioactive scaffolds, recombinant human growth factors (rhBMP-2, rhPDGF-BB), MSCs, and 3D-printed constructs. These innovations have enhanced alveolar bone regeneration, implant osseointegration, and periodontal tissue repair, offering predictable clinical outcomes. *Materials and Methods:* A review of the literature published between 2015 and 2025 was conducted through PubMed, Scopus, Web of Science, Embase, and Google Scholar. Clinical and preclinical studies on the use of CO_2_, Nd:YAG, Er:YAG, diode, and 445 nm lasers, biomaterials, PRP, MSCs, growth factors, and 3D-printed scaffolds were included. *Results:* Laser applications demonstrated significant benefits in epithelialization, biostimulation, and reduction in postoperative discomfort in soft tissues. For hard tissues, the combined use of MSCs, bioactive scaffolds, and growth factors promoted osteogenic differentiation, bone volume preservation, and improved mechanical stability. Photobiomodulation enhanced osteoblastic activity and accelerated bone remodeling, while 3D-printed scaffolds provided personalized architecture for optimal integration. *Conclusions:* Regenerative approaches integrating lasers, biomaterials, PRP, MSCs, growth factors, and 3D printing represent safe, minimally invasive, and effective strategies for the regeneration of both soft and hard oral tissues. These multidisciplinary techniques improve healing quality, functional recovery, and esthetic outcomes, reflecting the growing trend toward precision and technology-driven regenerative oral surgery.

## 1. Introduction

Soft tissue regeneration in oral surgery is essential for the functional and esthetic success of procedures such as tooth extraction, periodontal therapy, and implant placement. Delayed or impaired healing can lead to infection, complications, and poor esthetic outcomes, especially in the anterior maxilla [1,2]. In recent years, emphasis has been placed on using integrated laser therapy, biomaterials, platelet-rich plasma (PRP), mesenchymal stem cells (MSCs), and 3D-printed scaffolds to boost soft tissue repair [1,2,3,4,5,6,7,8,9,10]. These multimodal strategies produce synergistic effects that speed up healing, enhance tissue quality, and lower complications. Parallel to advances in soft tissue, hard tissue regeneration—such as alveolar bone and maxillofacial reconstruction—is vital for implant stability and esthetic restoration after trauma or resection [11,12,13,14,15,16,17,18]. Traditional grafts are increasingly complemented by bioactive scaffolds that mimic bone properties while supporting osteoconduction and osteoinduction [11,15,16,17]. Modern 3D-printed scaffolds offer precise fit, porosity, and controlled degradation aligned with bone formation, often functionalized with [8,9,10]. MSCs and growth factors to improve osteogenic potential [8,10,11,12]. Emerging trends include smart biomaterials with controlled growth factor release, bioprinted cell-laden matrices, and stem cell–derived secretome therapies, marking a shift from passive grafting to bioactive, patient-specific regenerative structures [10,11,12,13,14]. These advancements position hard tissue engineering as a crucial partner to soft tissue regeneration in full oral rehabilitation.

## 2. Materials and Methods

### 2.1. Focused Question

What are the current regenerative surgical methods, their biological and technological foundations, and what prospects are emerging for the clinical use of regenerative medicine in surgery?

### 2.2. Eligibility Criteria

The following inclusion criteria guided the analysis of the studies: (I) study design—narrative reviews, systematic reviews, meta-analyses, randomized controlled trials; (II) participants—human models; (III) interventions—oral surgical procedures to regenerate oral soft tissues and bone in the oral cavity; (IV) outcomes—latest strategies for regenerative surgery techniques and clinical management indications. We considered including only studies with free full texts available. Only studies that met all the inclusion criteria were included. It was also considered to exclude (I) abstracts of articles published in non-English languages, (II) duplicate studies, (III) in vitro or animal clinical studies, (IV) not pertinent studies, and (V) the absence of free full texts.

### 2.3. Search Strategy

This narrative review was conducted using the PICO framework (Population, Intervention, Comparison, Outcome) to structure the research question and guide the literature search. A comprehensive search was performed in PubMed (MEDLINE), Scopus, Web of Science, Embase, and Google Scholar electronic databases to identify relevant studies. The search strategy focused on the Population and Intervention components, as recommended to optimize recall and avoid missing pertinent literature. Abstracts and full texts of studies evaluating clinical outcomes and surgical techniques in regenerative oral surgery were screened and reviewed for inclusion.

### 2.4. Research

The medical subject heading (MeSH) terms are oral surgery, regenerative oral surgery, bone regeneration, soft tissue regeneration, PRP, laser therapy, photobiomodulation, advanced biomaterials, platelet-rich plasma (PRP), mesenchymal stem cells (MSCs), three-dimensional (3D) printing technologies, and growth factor–based regenerative strategies. An electronic search was carried out with the PubMed (MEDLINE) Scopus and Web of Science, Embase, and Google Scholar databases. The articles published in the years 2015–2025 were targeted. Titles and abstracts of all retrieved articles were independently screened for relevance. Studies not meeting the inclusion criteria were excluded at this stage. The remaining articles were then assessed at the full-text level to confirm eligibility and to identify any additional relevant studies through reference list review. For data extraction, the full texts of all included studies were reviewed, and pertinent findings related to clinical outcomes and surgical techniques in regenerative oral surgery were systematically recorded using a standardized approach.

### 2.5. Results

The primary search identified 4915 articles based on MeSH terms, published from 2015 to 2025. Following those, the research has been restricted to narrative reviews, systematic reviews, and meta-analyses and randomized controlled trials concerning human studies and the English language, so 144 results were found. As a result, it was decided that only articles with full text or free full text should be used.

## 3. Results

### 3.1. Laser Therapy

Laser therapy has established itself as a central modality in oral soft and hard tissue regeneration due to its ability to provide precise surgical control, antimicrobial effects, and photobiomodulation. Various laser systems (445 nm diode, CO_2_, Nd:YAG, Er:YAG, and other diode lasers) have been applied with customized parameters based on tissue type and clinical objectives [1,2,3,4,5,6,7,8,9,10,19] (Table 1).

**Mechanisms of Action**

Laser therapy exerts both thermal and nonthermal effects. Thermal interactions allow for precise incision, coagulation, and sterilization of the surgical field. These biological effects underpin the regenerative role of laser therapy in both soft and hard tissue [7,8,20].

**Soft Tissue Applications**

- **445 nm Diode** Laser: Several studies have reported faster epithelial healing and improved postoperative recovery after application of the 445 nm diode laser, supporting its use in minimally invasive soft tissue procedures.
- **CO_2_ Laser:** Clinical and preclinical evidence supports predictable soft tissue healing with preserved tissue architecture, reduced intraoperative bleeding, and favorable postoperative outcomes.
- **Nd:YAG and Diode Lasers**: These systems have demonstrated favorable healing responses and effective infection control in periodontal and peri-implant tissue, helping to improve postoperative stability.
- **Er:YAG Laser**: Use of the Er:YAG laser has been associated with effective wound closure and favorable gingival healing, particularly in procedures requiring minimal collateral thermal damage.

Overall clinical outcomes in soft tissue regeneration include accelerated wound closure, reduced intraoperative and postoperative bleeding, shorter healing times, improved soft tissue thickness and stability, and increased patient comfort [1,2,7,8,20].

**Hard Tissue Applications**

In hard tissue, photobiomodulation supports osteogenesis, angiogenesis, and bone remodeling through mechanisms consistent with those described for soft tissue regeneration. Clinically, photobiomodulation has been associated with improved alveolar ridge preservation after tooth extraction, accelerated bone healing, increased early implant stability, and improved long-term bone quality. These effects complement conventional regenerative approaches without replicating the underlying molecular mechanisms discussed previously [8,20,21,22] (Table 2).

### 3.2. Photobiomodulation and Oral Tissues

While the primary focus of this review centers on surgical wound healing, the inclusion of broader photobiomodulation (PBM) studies significantly strengthens the mechanistic foundation underlying oral tissue regeneration. Recent evidence demonstrates that PBM activates coordinated signaling networks through a redox-mediated NFκB-TGF-β1-ATF-4 axis, which modulates inflammation, adaptive stress responses, and tissue healing in oral keratinocytes [23]. These fundamental mechanisms extend beyond surgical applications to encompass mucosal health restoration, as evidenced by meta-analyses showing PBM’s efficacy in treating chemotherapy-induced oral mucositis through reduction in inflammatory cytokines (IL-1β, IL-18) and upregulation of anti-inflammatory mediators (IL-10) [24,25,26,27]. The biological basis for these effects involves photon absorption by cytochrome c oxidase in the mitochondrial respiratory chain, leading to increased ATP synthesis, activation of growth factor secretion (including TGF-β1, VEGF, and FGF), and modulation of multiple signaling pathways (PI3K/AKT, MAPK, NF-κB) that promote cellular proliferation, migration, and differentiation [24,25,26,27]. Furthermore, PBM demonstrates significant effects on neurosensory restoration through enhanced expression of neurotrophic factors (BDNF, NGF), myelin basic protein, and laminin, which are critical for peripheral nerve regeneration following trigeminal nerve injuries [24,25,26,27]. These mechanisms collectively support long-term tissue homeostasis by promoting macrophage polarization toward anti-inflammatory M2 phenotypes, enhancing neoangiogenesis through HIF-1α and VEGF upregulation, and facilitating extracellular matrix remodeling via modulation of matrix metalloproteinases [8,9,10].

### 3.3. Advanced Biomaterials and Platelet-Rich Plasma (PRP)

#### 3.3.1. Biomaterials

Advanced biomaterials serve as both structural and biochemical scaffolds that support cellular adhesion, proliferation, differentiation, and extracellular matrix deposition [2,3,6,11,12,14]. They have been extensively studied in oral soft and hard tissue regeneration, with natural and synthetic materials demonstrating efficacy in enhancing healing outcomes [2,3,6,13,14,15] (Table 3).

***Soft Tissue Biomaterials:***

- Collagen-based scaffolds: Promote fibroblast attachment, epithelial proliferation, and angiogenesis. Studies report improved epithelialization, faster wound closure, and increased gingival thickness when used in combination with soft tissue grafts [2,3].
- Chitosan-based scaffolds: Enhance cell adhesion and tissue integration. Preclinical studies demonstrate accelerated mucosal healing and improved vascularization [2,3].
- Bioactive polymers and ceramics: Provide structural support and maintain tissue volume. Clinical evidence indicates enhanced graft stability, improved soft tissue density, and reduction in post-surgical shrinkage [3,6].
- Functionalized scaffolds: Incorporation of growth factors such as PDGF and BMP within scaffolds further enhances regenerative potential, stimulating angiogenesis and extracellular matrix deposition [6].

***Hard Tissue Biomaterials:***

- Calcium phosphate ceramics and bioactive glass scaffolds: Promote osteoconduction and osteoinduction, supporting new bone formation and defect filling [2,11,17].
- Polymeric scaffolds (PLGA, PCL): Provide a biodegradable framework for mesenchymal stem cells (MSCs) and growth factors, improving cell retention and bone regeneration [15,16].
- Stem cell-loaded scaffolds: MSCs seeded on scaffolds enhance osteogenic differentiation, mineralized matrix deposition, and bone volume restoration in alveolar defects and extraction sockets [1,11,17].
- Growth factor-functionalized scaffolds: Incorporation of rhPDGF-BB, rhBMP-2, or other bioactive molecules stimulates angiogenesis and accelerates bone maturation, resulting in improved bone density and volume maintenance [5,11,17].

Numbered outcomes for biomaterials:

1. Enhanced epithelialization, angiogenesis, and osteogenesis across multiple animal models and clinical trials [2,3,6,11,17].
2. Increased soft tissue thickness and stability, with reduced postoperative volume loss [2,3].
3. Improved predictability of regenerative outcomes when biomaterials are combined with PRP or MSCs [2,4,6,11,17].
4. Accelerated wound closure and improved integration with surrounding native tissues, including bone [3,6,11].

#### 3.3.2. Platelet-Rich Plasma (PRP)

Platelet-rich plasma (PRP) is an autologous blood derivative enriched in platelet-associated growth factors and has been widely applied in oral regenerative surgery as an adjunctive therapy [4,5]. Rather than acting as a stand-alone regenerative solution, PRP primarily enhances the biological performance of biomaterials and cellular therapies, improving early healing dynamics and clinical predictability [4,5,6].

**Soft tissue effects**

Clinically, PRP application has been associated with accelerated wound healing, increased gingival thickness, and reduced postoperative pain and edema [4,5]. When combined with biomaterial scaffolds, PRP improves graft integration, stability, and early vascularization, contributing to more predictable soft tissue regenerative outcomes [4,6].

**Hard tissue effects**

In hard tissue regeneration, PRP use has been correlated with faster bone defect healing, improved early bone fill, and better preservation of alveolar ridge volume [1,4,5,11,17]. When applied in combination with scaffolds or mesenchymal stem cells, PRP supports early implant stability and enhances the overall predictability of bone regenerative procedures [5,6,11,17].

Overall, the regenerative benefits of PRP are most evident when it is employed as part of a multimodal strategy integrating biomaterials, cellular therapies, or laser-assisted approaches, rather than as a monotherapy [5,6,11,17,28,29,30,31,32,33,34,35,36,37,38,39].

### 3.4. Mesenchymal Stem Cells (MSCs)

#### Sources and Mechanisms

Mesenchymal stem cells (MSCs) derived from adipose tissue, bone marrow, and dental pulp play a pivotal role in oral soft and hard tissue regeneration due to their multipotent differentiation capacity and immunomodulatory activity [1,11,12,16,18,19]. Their primary contribution to regeneration relies on their ability to support tissue repair through differentiation into site-specific cell lineages and through paracrine signaling that promotes tissue homeostasis and integration.

Adipose-derived MSCs have demonstrated particular efficacy in soft tissue regeneration, where they enhance gingival thickness, support epithelial repair, and improve vascularization, making them suitable for mucosal augmentation and wound healing procedures in oral surgery [1,2,3,4,5,19]. Bone marrow-derived MSCs are considered the reference standard for hard tissue regeneration, owing to their robust osteogenic differentiation potential and their ability to support extracellular matrix deposition and angiogenesis in bone defects [1,5,11,17]. Dental pulp-derived MSCs exhibit high proliferative capacity and favorable tissue integration, supporting mucosal healing and connective tissue regeneration with a low risk of fibrotic response, making them particularly relevant for periodontal and oral mucosal applications [5,6,11,17,18].

Although each MSC source presents specific advantages depending on the clinical indication, all MSC populations share common regenerative properties that make them suitable for oral and maxillofacial regenerative strategies [1,11,12,16,18,19,36] (Table 4).

**Hard tissue sources and effects:**

- Bone marrow-derived MSCs: Promote osteoblast differentiation, extracellular matrix mineralization, and angiogenesis, leading to improved bone volume and alveolar ridge preservation [1,11,17].
- Dental pulp-derived MSCs: Support dentin-pulp complex regeneration and accelerate bone defect healing when combined with scaffolds or growth factors [11,17,18].
- Stem cell-seeded scaffolds: MSCs loaded onto calcium phosphate, bioactive glass, or polymeric scaffolds enhance osteoconduction, osteoinduction, and mineralized matrix deposition in preclinical and clinical studies [11,15,16,17].

**Hard Tissue Application**

In hard tissue regeneration, MSCs contribute to osteogenesis, mineralized matrix deposition, and bone volume preservation when applied alone or in combination with scaffolds and growth factors [1,11,17]. Bone marrow- and dental pulp-derived MSCs have demonstrated efficacy in alveolar ridge preservation, bone defect repair, and peri-implant regeneration, particularly when delivered through bioactive or polymeric scaffolds that enhance cell retention and differentiation [11,15,16,17]

**Clinical Outcomes**

Clinically, MSC-based therapies have been associated with increased soft tissue thickness, improved epithelialization, enhanced vascularization, and reduced fibrotic healing responses in oral soft tissues [1,5,6]. In hard tissues, MSC application supports accelerated bone formation, improved defect repair, and enhanced structural stability of regenerated bone [1,11,17,40,41,42,43,44,45].

The regenerative efficacy of MSCs is further enhanced when combined with biomaterials, platelet-rich plasma, or laser-assisted photobiomodulation. These multimodal approaches improve cell survival, promote tissue remodeling, and result in more predictable regenerative outcomes across both soft and hard tissue applications [1,6,11,17,21,22].

### 3.5. Three-Dimensional Printing

#### Scaffold Design and Customization

3D printing enables the fabrication of patient-specific scaffolds tailored to individual anatomical defects, integrating bioactive biomaterials, growth factors, and MSCs to optimize oral soft and hard tissue regeneration [1,2,11,15,17,19].

Customized scaffold geometry improves graft adaptation, stability, and osteoco dotion, ensuring optimal integration with surrounding tissues [11,15,16].

Integration with MSCs, PRP, or growth factors (PDGF, BMP) promotes angiogenesis, epithelialization, connective tissue formation, and osteogenesis, enhancing both soft and hard tissue healing [1,5,11,17].

Patient-specific designs facilitate minimally invasive procedures, improve defect-specific fit, and support reproducible regenerative outcomes in clinical practice [2,8,9,15,40,41,42,43] (Table 5).

Numbered outcomes:

- Accelerated epithelial and connective tissue formation, with enhanced soft tissue healing compared with conventional grafting [1,9].
- Enhanced vascularization, tissue density, and mineralized matrix deposition in bone defects [11,17].
- Better volumetric stability and long-term maintenance of regenerated soft and hard tissue [2,11,16].
- Predictable and reproducible outcomes suitable for clinical translation, including alveolar ridge preservation and dental implant site preparation [1,8,15].

Numbered outcomes:

1. Superior healing rates and tissue integration compared to conventional procedures [8,9].
2. Reduced postoperative complications and improved recovery [8].
3. Enhanced esthetic results through tailored scaffold designs [9].
4. Scalable and reproducible methods for clinical application [8,9].

## 4. Discussion

The integration of lasers, advanced biomaterials, platelet-rich plasma (PRP), mesenchymal stem cells (MSCs), and three-dimensional (3D) printing technologies represents a significant advancement in the field of oral tissue regeneration, encompassing both soft and hard tissues, as highlighted by a growing body of preclinical and clinical studies conducted over the last decade [1,2,3,4,5,6,7,8,9,10,11,12,13,14,15,16,17,18,21,22]. Lasers, including CO_2_, Nd:YAG, Er:YAG, diode, and the recently investigated 445 nm diode wavelength, provide a multifaceted contribution to tissue healing. In soft tissues, laser-assisted procedures reduce intraoperative and postoperative bleeding, minimize patient-reported pain, accelerate epithelialization, and improve predictability of wound healing outcomes [1,2,3,7,8,9,10,46,47,48]. Photobiomodulation induced by lasers stimulates cellular proliferation, angiogenesis, and activation of resident stem cells in the periodontal ligament and surrounding connective tissues, thereby creating a regenerative microenvironment [7,9,10]. The 445 nm diode laser specifically accelerates epithelialization, reduces inflammatory cell infiltration, and enhances the structural organization of newly regenerated epithelium [3,20,44,45,46,47,48].

In hard tissues, lasers have been shown to promote osteoblastic differentiation, enhance bone matrix deposition, and accelerate alveolar ridge preservation after tooth extraction, as well as improve peri-implant bone healing [7,21,22]. Clinical studies report that low-level laser therapy can enhance early bone formation, reduce postoperative inflammation, and improve bone density and implant stability, thereby complementing conventional bone grafting procedures [21,22]. Mechanistically, laser-induced photobiomodulation in hard tissues upregulates growth factors such as VEGF, PDGF, TGF-β, and BMPs, stimulating osteogenesis, angiogenesis, and extracellular matrix deposition within bone [22,49,50,51,52,53,54]. The combination of soft and hard tissue laser applications allows simultaneous optimization of mucosal and osseous healing, particularly in sites requiring both gingival and alveolar bone regeneration [8,21].

Advanced biomaterials provide structural and biochemical scaffolds essential for tissue regeneration. Natural polymers (collagen, chitosan, hyaluronic acid) and synthetic bioactive polymers and ceramics support cellular adhesion, proliferation, differentiation, and extracellular matrix deposition [2,3,6,11,15,18]. Scaffold architecture, including porosity, mechanical properties, and biodegradability, significantly influences cellular infiltration, vascularization, and osteoconduction [2,3,11,15]. Functionalization with bioactive molecules such as PDGF, BMPs, TGF-β, and VEGF further enhances both soft tissue and bone regeneration by modulating angiogenesis, inflammation, and osteogenic differentiation [3,5,6,11,17]. PRP acts synergistically with scaffolds in both soft and hard tissue contexts, accelerating epithelial and connective tissue formation, improving vascularization, and enhancing early bone regeneration when combined with graft materials or MSCs [4,5,11,17].

MSCs play a pivotal role in orchestrating regenerative processes across tissue types. Derived from adipose tissue, bone marrow, or dental pulp, MSCs exert multipotent differentiation potential and immunomodulatory effects, contributing to fibroblast, endothelial, and osteoblast lineages while secreting paracrine factors that stimulate angiogenesis, recruit progenitor cells, and limit fibrotic responses [1,5,6,11,17]. In soft tissue regeneration, MSCs enhance epithelialization, connective tissue formation, vascularization, and tissue thickness, with superior structural organization when combined with biomaterials or PRP [1,5,6]. In hard tissue regeneration, MSCs contribute to osteogenesis, scaffold integration, bone volume maintenance, and accelerated bone healing in alveolar defects or extraction sockets [11,16,17]. Clinical studies report that MSC-based therapies, particularly when combined with bioactive scaffolds or PRP, improve both soft and hard tissue outcomes, ensuring volumetric stability, long-term functionality, and enhanced graft survival [1,5,11,17,55,56,57,58,59,60].

Three-dimensional printing technologies further advance tissue engineering by enabling patient-specific scaffolds for both soft and hard tissues. Customized 3D scaffolds facilitate precise adaptation to defect morphology, controlled porosity, and integration of MSCs, PRP, and growth factors, thereby optimizing vascularization, epithelialization, and osteogenesis [8,9,11,15]. Clinical evidence indicates that 3D-printed constructs improve graft adaptation, reduce operative time, and allow minimally invasive procedures while maintaining reproducible regenerative outcomes [8,9]. The combination of 3D-printed scaffolds with laser therapy offers additional benefits, including local photobiomodulation to enhance angiogenesis, reduce inflammation, accelerate epithelial closure, and stimulate osteoblastic activity [40,41,42,43,44,45].

***Limitation of the study and future research.***

Multimodal regenerative strategies integrating lasers, biomaterials, PRP, mesenchymal stem cells, and 3D printing demonstrate superior efficacy compared to monotherapies, thanks to synergistic action on cellular, molecular, and mechanical processes, with documented improvements in soft and hard tissue regeneration and greater clinical predictability. However, the strong heterogeneity of experimental and clinical protocols, regarding laser parameters, PRP preparation, scaffold characteristics, and MSC use modalities, limits the comparability and reproducibility of results. Furthermore, the long-term stability and functional integration of regenerated tissues remain poorly explored. Future perspectives include conducting multicenter clinical trials with standardized protocols and the development of advanced biomaterials, bioprinting technologies, and intelligent laser systems, with the aim of further optimizing and personalizing regenerative strategies in oral and maxillofacial surgery.

## 5. Conclusions

Oral reconstructive surgery is gradually evolving toward an integrated strategy that combines surgical precision and biostimulation. Based on a summary of the reviewed literature, the combined use of laser technology, advanced biomaterials, platelet-rich plasma, mesenchymal stem cells, and 3D printing appears to improve soft and hard tissue healing compared to conventional methods. Overall, these combined approaches reflect a shift toward personalized and minimally invasive reconstructive surgery. However, the variability of clinical protocols underscores the need for continued longitudinal and standardized clinical studies to better define indications and optimize treatment strategies.

## Figures and Tables

**Table 1:** This table summarizes the most used laser types in oral soft tissue regeneration, highlighting their mechanisms of action and observed clinical or preclinical outcomes. Diode lasers at 445 nm are particularly effective for accelerating epithelialization and modulating inflammation, while CO_2_, Nd:YAG, and Er:YAG lasers provide precise surgical control, hemostasis, and enhanced fibroblast activity. Photobiomodulation further supports stem cell activation, contributing to tissue remodeling.

Laser Type	Mechanism	Clinical/Preclinical Outcomes	References
445 nm diode	Biostimulation, enhanced epithelialization	Accelerates epithelialization, reduces inflammation, stimulates fibroblast proliferation	[3,10,20]
CO_2_	Precise cutting, hemostasis	Reduces intraoperative bleeding, preserves extracellular matrix, promotes faster wound closure	[1,2,7]
Nd:YAG	Deep tissue penetration, antimicrobial	Reduces bacterial load, modulates inflammation, enhances fibroblast activity	[7,8]
Er:YAG	Selective ablation, minimal thermal damage	Efficient wound closure, preserves gingival architecture, enhances epithelialization	[7,8]
Diode	Biostimulation, decontamination	Faster healing, reduced pain, activation of resident stem cells	[7,9]

**Table 2:** This table summarizes the most used laser types in oral hard tissue regeneration, highlighting their mechanisms of action and observed clinical or preclinical outcomes. Diode and Nd:YAG lasers promote osteoblast proliferation, angiogenesis, and antimicrobial effects, while CO_2_ and Er:YAG lasers allow precise bone ablation, hemostasis, and stimulation of extracellular matrix deposition. Photobiomodulation enhances osteogenic activity and bone remodeling, contributing to accelerated healing and improved bone-to-implant integration.

Laser Type	Mechanism	Clinical/Preclinical Outcomes	References
Low- level diode laser (LLLT)	Stimulates osteoblast proliferation and differentiation via MAPK/ERK and Wnt/β-catenin pathways; enhances matrix mineralization; promotes angiogenesis; anti-inflammatory and antimicrobial effects	Accelerated bone healing; improved trabecular organization; increased bone-to-implant contact; reduced alveolar ridge resorption; enhanced implant stability; faster radiographic bone fill	[7,8,21,22]
CO_2_	Precise cutting with minimal thermal damage; photobiomodulation promotes osteogenic differentiation; improves local vascularization	Reduces intraoperative bleeding, preserves extracellular matrix, promotes faster wound closure	[7,8,22]
Nd:YAG	Deeper tissue penetration; antimicrobial and anti-inflammatory; stimulates osteogenic signaling and vascularization	Reduces bacterial load, modulates inflammation, enhances fibroblast activity	[7,8,22]
Er:YAG	Selective ablation of hard tissue; promotes osteoblast activity and extracellular matrix deposition; supports angiogenesis	Efficient wound closure, preserves gingival architecture, enhances epithelialization	[7,8,22]
Laser + PRP/MSCs	Synergistic effect combining photobiomodulation with growth factors and stem cells; boosts osteogenic differentiation and angiogenesis	Faster healing, reduced pain, activation of resident stem cells	[1,4,21,22]

**Table 3:** This table integrates advanced biomaterials, PRP, MSCs, and laser therapies for oral soft and hard tissue regeneration. Collagen, chitosan, and bioactive polymers enhance soft tissue healing, while calcium phosphate, bioactive glass, and polymeric scaffolds support bone regeneration. PRP delivers autologous growth factors to accelerate angiogenesis and matrix deposition in both tissue types. Stem cell-loaded and growth factor-functionalized scaffolds further enhance osteogenesis. Laser therapies, including diode, CO_2_, Nd:YAG, and Er:YAG, accelerate epithelial and bone healing through photobiomodulation and cellular stimulation. Combined approaches yield synergistic and predictable regenerative outcomes across preclinical and clinical studies.

Material/Therapy	Mechanism	Clinical/Preclinical Outcomes	References
Collagen scaffolds (soft tissue)	Promote fibroblast adhesion, epithelial proliferation, angiogenesis	Improved epithelialization, faster wound closure, increased gingival thickness	[2,3]
Chitosan scaffolds (soft tissue)	Enhances cell adhesion and vascularization	Accelerated mucosal healing, better tissue integration	[2,3]
Bioactive polymers (soft tissue)	Provide structural support and maintain tissue volume	Enhanced graft stability, improved soft tissue density, reduced postoperative shrinkage	[3,6]
Functionalized scaffolds with growth factors (soft tissue)	Stimulates angiogenesis and ECM deposition	Superior epithelialization, improved soft tissue regeneration	[6]
Platelet-Rich Plasma (PRP) (soft tissue and hard tissue)	Rich in growth factors (PDGF, VEGF, TGF-β, BMPs) important for soft tissue In hard tissue promotes osteoblast proliferation, angiogenesis, mineralized matrix deposition	Faster wound healing, increased tissue density, synergistic effects with MSCs and biomaterials Faster bone defect healing, improved bone volume and implant stability	[1,4,5,11,17]
Calcium phosphate/bioactive glass scaffolds (hard tissue)	Osteoconduction and osteoinduction	New bone formation, defect filling, alveolar ridge preservation	[2,11,17]
Polymeric scaffolds (PLGA, PCL) (hard tissue)	Biodegradable framework for MSCs and growth factors	Enhanced cell retention, osteogenic differentiation, mineralized matrix deposition	[15,16]
Stem cell-loaded scaffolds (hard tissue)	MSCs enhance osteogenesis and matrix deposition	Increased bone volume, defect repair, improved implant stability	[1,11,17]
Growth factor-functionalized scaffolds (hard tissue)	rhPDGF-BB, rhBMP-2 stimulate angiogenesis and osteogenesis	Accelerated bone maturation, improved bone density and volume maintenance	[5,11,17]
Combination therapy (biomaterials + MSCs + PRP + laser)	Synergistic effects: scaffolds support cells, PRP provides growth factors, laser enhances cell activity	Optimized regenerative outcomes, predictable soft and hard tissue healing, improved structural and functional restoration	[1,2,3,4,5,6,11,17,19,21,22]

**Table 4:** MSCs from various sources are used to promote angiogenesis, proliferation, and connective tissue formation and stability. Combining MSCs with PRP or laser therapy results in synergistic effects, improving epithelialization, vascularization, and overall tissue quality, while minimizing fibrosis and accelerating recovery.

MSC Source	Mechanism	Clinical/Preclinical Outcomes	References
Adipose tissue (soft tissue)	Angiogenesis, epithelial proliferation	Improved gingival thickness, enhanced vascularization, faster healing	[1,6]
Bone marrow	In soft tissue immunomodulation, collagen deposition, angiogenesis; differentiation into fibroblasts In hard tissue osteogenic differentiation, VEGF-mediated angiogenesis; extracellular matrix mineralization	Enhanced connective tissue formation, extracellular matrix remodeling, Enhanced bone formation, alveolar ridge preservation, improved bone density in defects	[1,5,11,17]
Dental pulp	In soft tissue–paracrine signaling; stimulate epithelial and connective tissue regeneration In hard tissue: Support dentin-pulp complex regeneration; osteoblast differentiation	Rapid epithelialization, improved tissue architecture	[5,6,11,17,18]
MSC + PRP	Stimulates angiogenesis and ECM deposition	Enhanced epithelialization and connective tissue formation, faster healing	[5,6,11,17]
MSC + Laser	Biostimulation + stem cell activation	Improved tissue remodeling, reduced fibrosis, increased tissue density	[1,6,10,21,22]

**Table 5:** This table summarizes the role of 3D-printed, patient-specific scaffolds in oral tissue regeneration, highlighting both soft and hard tissue applications. Customized scaffold geometry ensures optimal graft adaptation, volumetric stability, and reproducible regenerative outcomes. Integration with MSCs, PRP, or growth factors such as PDGF and BMP enhances angiogenesis, epithelialization, connective tissue formation, and bone regeneration. These strategies accelerate healing, improve tissue density, and support long-term maintenance of regenerated tissues, making them highly suitable for clinical.

MSC Source	Mechanism	Clinical/Preclinical Outcomes	References
3D-printed scaffold	Patient-specific design improves graft adaptation and stability; supports both soft tissue integration and bone conduction	Improved graft stability, faster epithelialization	[2,8,9,11,15,16]
Scaffold + MSCs	MSCs provide paracrine factors, immunomodulation, and differentiation into fibroblasts/osteoblasts; promote angiogenesis, epithelialization, connective tissue formation, and osteogenesis	Enhanced connective tissue formation, increased vascularization	[1,5,11,17]
Scaffold + PRP	Growth factors stimulate angiogenesis, collagen deposition, extracellular matrix formation, and mineralized bone matrix production	Faster wound closure, increased tissue density	[1,5,11,17,22]
Scaffold + Laser	Biostimulation and cellular activation	Improved tissue remodeling, predictable regenerative outcomes	[8,9]

Translation in complex oral and maxillofacial defects.

## Data Availability

Upon request to the corresponding author, the data are available for use.
